# Supplementary material for: National priority setting partnership using a Delphi consensus process to develop neonatal research questions suitable for practice-changing randomised trials in the United Kingdom
Source: BMJ Open. 2022 Sep 28;12(9):e061330. doi: 10.1136/bmjopen-2022-061330 (PMC9528679; doi:10.1136/bmjopen-2022-061330)
Supplement: Author's manuscript [file bmjopen-2022-061330.draft_revisions.pdf]

BMJ Open

BMJ Open is committed to open peer review. As part of this commitment we make the peer review history of every article we publish publicly available.

When an article is published we post the peer reviewers' comments and the authors' responses online. We also post the versions of the paper that were used during peer review. These are the versions that the peer review comments apply to.

The versions of the paper that follow are the versions that were submitted during the peer review process. They are not the versions of record or the final published versions. They should not be cited or distributed as the published version of this manuscript.

BMJ Open is an open access journal and the full, final, typeset and author-corrected version of record of the manuscript is available on our site with no access controls, subscription charges or pay-per-view fees (http://bmjopen.bmj.com).

If you have any questions on BMJ Open's open peer review process please email info.bmjopen@bmj.com

BMJ Open

Protocol: A national priority setting partnership to develop neonatal research questions suitable for practice-changing randomised trials in the United Kingdom.

Journal:	*BMJ Open*
Manuscript ID	bmjopen-2022-061330
Article Type:	Protocol
Date Submitted by the Author:	28-Jan-2022
Complete List of Authors:	Evans, Katie; Imperial College London Faculty of Medicine,<br>Battersby, Cheryl; Imperial College London, NHS England<br>Boardman, James; The University of Edinburgh, MRC Centre for Reproductive Health<br>Carroll, William; University Hospitals of North Midlands NHS Trust, Academic Department of Child Health; Keele University, Institute of Applied Clinical Sciences<br>Dinwiddy, Kate; British Association of Perinatal Medicine<br>Dorling, Jon; University Hospital Southampton NHS Foundation Trust<br>Gallagher, Katie; King's College London, Child and Adolescent Health<br>Hardy, Pollyanna; University of Oxford, Clinical Trials<br>Johnston, Emma; Parents and Families engagement lead at Thames Valley and Wessex Operational Deliveries Network<br>Mactier, Helen; University of Glasgow<br>Marcroft, Claire; Newcastle Upon Tyne Hospitals NHS Foundation Trust<br>Webbe, James; Imperial College London, Section of Neonatal Medicine<br>Gale, Chris; Imperial College London, Neonatal Medicine, School of Public Health ;
Keywords:	NEONATOLOGY, Protocols & guidelines < HEALTH SERVICES ADMINISTRATION & MANAGEMENT, Neonatal intensive & critical care < INTENSIVE & CRITICAL CARE

SCHOLARONE™ Manuscripts

Protocol: A national priority setting partnership to develop neonatal research questions suitable for practice-changing randomised trials in the United Kingdom.

Authors:

Katie Evans, BSc (hons), MBBS, MRCPCH. Honorary research fellow, Neonatal Medicine, Imperial College London. E: katie.evans7@nhs.net.

Cheryl Battersby, PhD. Neonatal Medicine, Imperial College London, School of Public Health, Faculty of Medicine. E: c.battersby@imperial.ac.uk.

James P. Boardman, PhD, FRCPCH. Professor of Neonatal Medicine, MRC Centre for Reproductive Health, University of Edinburgh, UK. E: james.boardman@ed.ac.uk.

William Carroll, MD, FRCPCH. Honorary Reader in Child Health, Keele University, Keele, UK. E: will.carroll@nhs.net.

Kate Dinwiddy, BA (hons). Chief Executive of British Association of Perinatal Medicine. E: kate.dinwiddy@rcpch.ac.uk.

Jon Dorling, MBChB, MD, FRCPCH. Department of Neonatal Medicine. Consultant Neonatology and Adjunct Professor. University Hospital Southampton NHS Foundation Trust, Southampton, UK. E: jon.dorling@uhs.nhs.uk.

Katie Gallagher, PhD, RN (child), Senior Research Fellow, EGA Institute for Women's Health, University College London, UK. E: katie.gallagher@ucl.ac.uk.

Pollyanna Hardy, MSc. Director of the National Perinatal Epidemiology Unit Clinical Trials Unit, University of Oxford, UK. E: Pollyanna.hardy@npeu.ox.ac.uk.

Emma Johnston, BA (hons), DipM. Parents and Family engagement Lead with the Thames Valley and Wessex Operational Delivery Network, UK. E: emma_jj@outlook.com.

Helen Mactier, MBChB, MD, FRCPCH. Consultant Neonatologist and Honorary Clinical Associate Professor, University of Glasgow. E: helen.mactier@glasgow.ac.uk.

For peer review only

For peer review only - http://bmjopen.bmj.com/site/about/guidelines.xhtml

Claire Marcroft, Clinical Specialist Neonatal Physiotherapist and NIHR CDRF Fellow, Newcastle upon Tyne Hospitals NHS Foundation Trust.

James Webbe, PhD, Neonatal Medicine, School of Public Health, Faculty of Medicine, Imperial College London. E: j.webbe@imperial.ac.uk.

Chris Gale, PhD, Reader in Neonatal Medicine, Imperial College London, School of Public Health, Faculty of Medicine, Chelsea and Westminster campus, 4th Floor, Lift Bank D, 369 Fulham Road, SW10 9NH, UK; Telephone: 0203 3153519; E: christopher.gale@imperial.ac.uk (corresponding author)

For peer review only

Abstract:

Introduction. Methodologically robust clinical trials are required to improve neonatal care and reduce unwanted variations in practice. Previous neonatal research prioritisation processes have identified important research themes rather than specific research questions amenable to clinical trials. Practice-changing trials require well defined research questions, commonly organised using the Population, Intervention, Comparison, Outcome (PICO) structure. By narrowing the scope of research priorities to those which can be answered in clinical trials and by involving a wide range of different stakeholders, we aim to provide a robust and transparent process to identify and prioritise research questions answerable within the NHS to inform future practice-changing clinical trials.

Methods and Analysis. A steering group comprising parents, doctors, nurses, allied health professionals, researchers, and representatives from key organisations (Neonatal Society (NS), British Association of Perinatal Medicine (BAPM), Neonatal Nurses Association (NNA) and Royal College of Paediatrics and Child Health (RCPCH)) was identified to oversee this project. We will invite submissions of research questions formatted using the PICO structure from the following stakeholder groups using an online questionnaire: parents, patients, healthcare professionals and academic researchers. Unanswered, non-duplicate research questions will be entered into a two round eDelphi survey of all stakeholder groups. Research questions will be ranked by mean aggregate scores.

Ethics and Dissemination. The final list of prioritised research questions will be disseminated through traditional academic channels, directly to key stakeholder groups through representative organisations and on social media. The outcome of the project will be shared with key research organisations such as the National Institute for Health Research (NIHR). Research ethics committee approval is not required.

Registration Details. Not registered.

For peer review only

Strengths and Limitations of this study:

- By involving parents and former patients alongside a wide range of healthcare professionals we will ensure that research questions are important to all key stakeholder groups.
- We will use established strategies (two round eDelphi process) to rank research questions based upon mean scores.
- The study will rank research questions based upon subjective input from a large number of key stakeholders, however questions may need further work prior to being addressed in a clinical trial.
- The final list of prioritised questions will be disseminated widely to inform neonatal clinical research.

For peer review only

Introduction:

The importance of involving different stakeholders including parents, patients, healthcare professionals and researchers to identify and prioritise research is well recognised[1]. In neonatal and perinatal medicine these stakeholder groups have prioritised research uncertainties in the fields of preterm birth[2,3], stillbirth[4], diabetes in pregnancy[5] and pregnancy hypertension[6]. Although these processes have been invaluable for prioritising research themes, the broad topics commonly identified have not been readily amenable to testing in randomised trials. Methodologically robust randomised controlled trials are the gold standard for assessing effectiveness of a healthcare intervention, drug or technology[7] and are critical to improving quality and reducing variation in neonatal care. Randomised clinical trials require clearly structured and focused research questions, which can be organised using the PICO model which guides the questioner to clearly identify the Participant (P), Intervention (I), Comparator (C) and Outcome (O)[8,9] (figure 1)[10].

There is wide variation in neonatal care[11,12] and an incomplete evidence base for many neonatal treatments,[13] consequently, there is a need to identify and prioritise research questions that can be tested in randomised trials. Involving all relevant stakeholders in such a process will ensure that research addresses questions that are important to healthcare professionals, former neonatal patients and parents, as well as relevant to current neonatal care.

The aim of this project is to identify and prioritise neonatal research questions in PICO format, suitable for practice-changing randomised trials, using a robust, transparent, and inclusive methodology. The results of this identification and prioritisation process will inform the development and design of neonatal randomised controlled trials in high income settings, including the National Healthcare System (NHS) in the United Kingdom.

Methods and Analysis

Steering Group

A steering group has been formed to agree the scope and facilitate the process of identifying and prioritising research questions. The following key stakeholders are represented on the steering group: the Neonatal Society (NS), British Association of Perinatal Medicine (BAPM), Neonatal Nurses Association (NA), Royal College of Paediatrics and Child Health, neonatal Allied Health Professionals (AHPs), neonatal clinical trial methodologists, neonatal trainees

For peer review only - http://bmjopen.bmj.com/site/about/guidelines.xhtml

and parents with experience of neonatal care. The steering group is co-chaired by representatives of the NS and BAPM. Details of the steering group members can be found in figure 2.

The roles of the steering group are as follows:

- Agreeing the scope of the process.
- Disseminating details of the process.
- Engaging with clinical stakeholders to take part in identifying and prioritising research questions.
- Review of submitted research questions to identify duplicate questions, questions that have already been answered and questions outside the scope.
- Disseminating the final ranked list of research questions.

Identification of stakeholders

The utility and validity of this project will depend on ensuring that representative questions are generated and prioritised by a wide group of neonatal stakeholders including:

- Clinicians involved in neonatal care: Neonatologists, paediatricians, neonatal nurses, advanced neonatal nurse practitioners (ANNPs) working in paediatrics or neonatal medicine. Recruitment will be through advertisements on the RCPCH website and through other relevant professional organisations including BAPM, the NNA and the NS. Trainee doctors and nurses will be additionally contacted through local training schools and use of communication methods such as regional teaching and social media channels.
- Allied Health Professionals: Occupational therapists, physiotherapists, speech and language therapists and clinical psychologists working in neonatal care: Advertisements will be placed through professional websites and organisations and co-ordinated by the AHP steering group representative.
- Academics and researchers working within neonatology: Recruitment will be targeted through academic organisations, existing research networks, national meetings and through Clinical Trials Units with a neonatal interest.
- Parents and former neonatal patients: These groups will be contacted through the National Care Coordinator Group, Maternity Voices partnerships, charity websites and through social media platforms.

For peer review only - http://bmjopen.bmj.com/site/about/guidelines.xhtml

To ensure maximal engagement across all stakeholder groups, various communications strategies will be employing including email contact, professional websites and social media routes.

Patient and Public Involvement

Parents and patients have been involved in this work from its inception. A parent representative sits on the steering group to plan the project and optimise parent and patient involvement. Communication to parents and former neonatal patients will be through parent and patient networks charities, regional support networks and social media groups. Parents and former neonatal patients will be involved in identifying neonatal research questions through the online identification process. To further facilitate the identification of research questions from parents and former neonatal patients we will develop targeted online support resources and run an online workshop focused on developing questions using a PICO format. Parents and former neonatal patients will then be involved in the prioritisation of research questions through the eDelphi process.

Scope

The scope of the prioritisation process has been agreed by the steering group as follows:

- Limited to research questions relevant to high income neonatal care settings.
- Limited to research questions related to care provided by neonatal teams:
  - On neonatal units, transitional care units, or as part of neonatal transport.
  - On postnatal wards (excluding care exclusively provided by midwifery teams without neonatal input).
  - In the community after receiving neonatal care as an inpatient (care may be provided by community neonatal teams or associated community allied health professionals with neonatal interest).

Overview

This study will be divided into four stages:

1. Identification of neonatal research questions suitable for analysis in randomised controlled trials.
2. Review of submitted neonatal research questions to remove duplicate questions and previously answered questions.

For peer review only - http://bmjopen.bmj.com/site/about/guidelines.xhtml

3. Prioritisation of neonatal research questions by all relevant stakeholders using a two-round eDelphi process.
4. Dissemination of ranked list of PICO questions.

Stage 1: Identification of testable neonatal research questions

We will identify neonatal research questions suitable for evaluation in practice changing randomised controlled trials through an open process. Individual stakeholders (neonatal clinicians, neonatal nurses, ANNPs, neonatal AHPs, neonatal researchers and former neonatal patients and patients) will be contacted via professional organisations, social media platforms, networks and organisational mailing lists. The process for identifying research questions will also be openly publicised on organisational websites seeking submission of research questions from stakeholders. Publicity will precede and continue throughout a four-week submission period[14] to optimise engagement and inclusion of all key groups.

Stakeholders will be invited to submit as many questions as they would like using an online system that facilitates submission using the PICO structure:

- Populations will be able to be selected from a pre-defined selection of gestations and clinical cohorts, or specified by the submitter
- Intervention and Comparisons will take the form of free text to allow full descriptions
- Outcomes will be able to be selected from Core Outcomes in Neonatology (COIN)[15], or specified by the submitter

Support in structuring research questions using PICO will be facilitated by the steering group members and organisations using techniques such as short videos. Contact details and basic demographic data will be requested from individuals who submit questions to monitor the representativeness of stakeholder involvement, and to invite participation in the subsequent prioritisation process.

Submission of relevant PICOs from previous priority setting work[16] will be welcomed.

Stage 2: Review and Refinement of the long-list of neonatal research questions

Submitted research questions will be reviewed by the steering group to remove duplicate, already answered and out of scope questions, and to refine those not consistent with the PICO

For peer review only - http://bmjopen.bmj.com/site/about/guidelines.xhtml

structure. Prior to exclusion, questions will be independently reviewed by two members of the steering group. A final long-list of research questions will be taken forward for prioritisation.

Stage 3: Prioritisation of Neonatal Research Questions

All research questions included in the long-list will be included in a two-round eDelphi prioritisation process. Involvement of stakeholders in the prioritisation process will be facilitated as follows:

- All stakeholders who submitted questions will be contacted by email and asked to take part.
- Invitations to take part will be circulated by professional organisations and open links will be made available on professional organisational websites and social media accounts.
- Parents and previous neonatal patients will be contacted through professional organisations and networks and through social media.

All research questions will be ranked using a 9-point Likert scale. After the first round there will be the opportunity to submit new PICO questions. At the second stage, stakeholders will be provided with information on how individual research questions were prioritised at the first stage.

After completion of the second round of ranking all research questions will be collated into a ranking list of research priorities for the UK neonatal community. Ranking will be ordered based upon mean score. Results will also be presented by mean score within each stakeholder group. All questions not excluded in the initial review process will be included in the final list.

Ethics and Dissemination:

Research Ethics Committee approval is not required for this work.

The ranked list of defined neonatal research questions will be disseminated as follows:

- We will share results directly with the NIHR prioritisation panel and other funders of neonatal trials.
- We will circulate the list to stakeholder organisations and their members.

For peer review only - http://bmjopen.bmj.com/site/about/guidelines.xhtml

- Individuals who participated in question setting or the prioritisation process will be emailed directly with the finalised rankings and named as group authors in any published work.
- Results will be disseminated amongst the scientific community through a publication and presented at relevant neonatal meetings.

Discussion:

More than 1 in 10 babies in many high-income settings will receive neonatal care. Neonatal conditions are important, contributing to almost half of all child deaths in the UK and to many long-term health conditions. Despite this importance, much neonatal care is not based upon high-quality research evidence. Historically, the perinatal research environment has proudly fostered collaborative working, demonstrated by studies such as EPICure[17]. The SARS-CoV-2 pandemic has further highlighted the benefits of joined up working across multiple geographical regions, scientific institutions and research groups in achieving practice changing outcomes within much shorter timeframes[18]. This has resulted in increased focus on prioritisation projects within healthcare using initiatives such as the James Lind Alliance to support unified decision making across specific specialities. Neonatology within the UK is ideally placed for such prioritisation work and future collaborative research due to centrally funded healthcare, the neonatal network structure facilitating close relationships between units and the accessibility of large national databases. Ultimately this process seeks to involve a wide range of key neonatal stakeholders, to identify and prioritise research questions addressing the many clinical uncertainties, suitable for evaluating in well-structured clinical trials.

Author Contributions:

CG and CB conceived this project. KE, JPB, HM, CG and CB planned and coordinated the initial steering group and protocol. The first draft of the manuscript was written by KE and revised by CB/ JPB/ WC/ JD/ KG/ PH/ EJ/ HM/ JW and CG. CG edited and reviewed the manuscript. It was approved by all members of the steering group.

Correspondence to Chris Gale, PhD, Reader in Neonatal Medicine, Imperial College London, School of Public Health, Faculty of Medicine, Chelsea and Westminster campus, 4th Floor,

For peer review only - http://bmjopen.bmj.com/site/about/guidelines.xhtml

Lift Bank D, 369 Fulham Road, SW10 9NH, UK; Telephone: 0203 3153519; E: christopher.gale@imperial.ac.uk.

Funding Statement:

Administrative support for the project is provided by BAPM; questionnaire and online Delphi software are funded by the Medical Research Council (MRC) through a Transition Support Award held by CG (MR/V036866/1).

Competing Interests Statement:

CG is vice chair of the NIHR Research for Patient Benefit London Regional Advisory Panel and a member of the Glasgow Children's Hospital Charity External Panel; he holds a Medical Research Council Transition Support Award.
JPB is a member of the Wellcome Trust's Career Development Award Panel and the Great Ormond Street Hospital Charity Research Assessment Panel.
CB is the NIHR deputy chair of HTA prioritisation committee for hospitals.
JD is a member of the NIHR HTA CET Funding Committee.
CM is funded by HEE-NIHR Integrated Clinical Academic Programme and holds a NIHR ICA CSRF Fellowship.

Acknowledgements:

We wish to thank the members of the Neonatal Priority Setting Partnership Steering Group for their support and RCPCH, BAPM, NNA and the NS for their support.

Word Count:

2334/ 4000 (including abstract/ excluding references)

References

1. Partridge N, Scadding J. The James Lind Alliance: patients and clinicians should jointly identify their priorities for clinical trials. *Lancet.* 2004; 364(9449): 1923-1924.

For peer review only

2. Duley L, Uhm S, Oliver S, Par PBPS. Top 15 UK research priorities for preterm birth. *Lancet.* 2014. 383(9934): 2041-2042.
3. Oliver S, Uhm S, Duley L, Crowe S, David AL, James CP, Chivers Z, Gyte G, Gale C, Turner M, Chambers B, Dowling I, McNeill J, Alderdice F, Shennan A & Deshpande S. Top research priorities for preterm birth: results of a prioritisation partnership between people affected by preterm birth and healthcare professionals. *BMC Pregnancy Childbirth.* 2019. 19(1): 528.
4. Heazell AEP, Whitworth MK, Whitcombe J, Glover SW, Bevan C, Brewin J, Calderwood C, Canter A, Jessop F, Johnson G, Martin I, Metcalf L. Research Priorities for Stillbirth: Process Overview and Results from the UK Stillbirth Priority Setting Partnership. *Ultrasound Obstetrics Gynaecology.* 2015. 46(6):641-647.
5. Ayman G, Strachan JA, McLennan N, Malouf R, Lowe-Zinola J, Magdi F, Roberts N, Alderdice F, Berneantu I, Breslin N, Byrne C, Carnell S, Churchill D, Grisoni J, Hirst JE, Morris A, Murphy HR, O'Brien J, Schmutz C, Shah K, Singal AS, Strachan MWJ, Cowan K, Knight M. The top 10 research priorities in diabetes and pregnancy according to women, support networks and healthcare professionals. *Diabetic Medicine.* 2021. 38(8):e14588.
6. Ho A, Webster L, Bowen L, Creighton F, Findlay S, Gale C, Green M, Gronlund T, Magee L, McManus RJ, Mistry HD, Singleton G, Thornton J, Whybrow R, Chappell L. Research priorities for pregnancy hypertension: a UK priority setting partnership with the James Lind Alliance. *BMJ Open.* 2020. 10:e036347.
7. Akobeng AK. Understanding randomised controlled trials. *Archives of Disease in Childhood.* 2005. 90:840-844.
8. Miller SA, Forrest JL. Enhancing your practice through evidence-based decision making: PICO, learning how to ask good questions. *Journal of Evidence Based Dental Practice.* 2001. 1: 136-141.
9. Richardson WS, Wilson MC, Nishikawa J, Hayward RS. The well-built clinical question: a key to evidence-based decisions. *ACP Journal Club.* 1995. 123(3): 12-13.
10. Curley A, Stanworth SJ, Willoughby K, Fustolo-Gunnink SF, Venkatesh V, Hudson C, Deary A, Hodge R, Hopkins V, Santamaria BL, Mora A, Llewelyn C, A-Amore A, Khan R, Onland W, Lopriore E, Fijnvandraat K, New H, Clarke P, Watts T on behalf of the PlaNeT2 MATISSE Collaborators. Randomized Trial of Platelet-Transfusion Thresholds in Neonates. *New England Journal of Medicine.* 2019. 380: 242-251.

For peer review only - http://bmjopen.bmj.com/site/about/guidelines.xhtml

11. National Neonatal Audit Programme (NNAP) 2020 annual report on 2019 data. RCPCH. London, 2020.
12. Pierrat V, Burguet A, Marchand-Martin L, Cambonie G, Coquelin A, Roze JC, Durox M, Guillois B, Morgan AS, Kaminski M on behalf of the Neurodevelopmental care study group of EPIPAGE-2. Variations in patterns of care across neonatal units and their associations with outcomes in very preterm infants: the French EPIPAGE-2 cohort study. *BMJ Open.* 2020. 10:e035075.
13. Willhelm C, Girisch W, Gottschling S, Graber S, Wahl H, Meyer S. Systematic Cochrane reviews in neonatology: a critical appraisal. *Paediatric Neonatology.* 2013. 54(4): 261-266.
14. Kost RG, Rosa JCd. Impact of survey length and compensation on validity, reliability and sample characteristics for Ultrashort-, Short-, and Long-Research Participant Perception Surveys. *Clinical Translational Science.* 2018. 2(1): 242-251.
15. Webbe J, Duffy JMN, Afonso E, Al-Muzaffar I, Brunton G, Greenough A, Hall NJ, Knight MK, Latour JM, Lee-Davey C, Marlow N, Noakes L, Nycyk J, Richard-Londt A, Wills-Eve B, Modi N, Gale C. Core outcomes in neonatology: development of a core outcome set for neonatal research. *Archives of Disease in Childhood - Fetal and Neonatal Edition.* 2020. **105:** 425-431.
16. Johnson MJ, Lapillonne A, Bronsky J, Domellof M, Embleton N, Iacobelli S, Jochum F, Joosten K, Kolacek S, Mihatsch WA, Moltu SJ, Puntis JWL, Riskin A, Shamir R, Tabbers MM, Goudoever JBV, Pipaon MSd on behalf of the ESPGHAN/ ESPEN/ ESPR/ CSPEN Working Group on Pediatric Parenteral Nutrition. Research priorities in pediatric parenteral nutrition: a consensus and perspective from ESPGHAN/ ESPEN/ ESPR/ CSPEN. *Pediatric Research.* 2021. Epub ahead of print: doi:10.1038/s41390-021-01670-9.
17. Moore T, Hennessy EM, Myles J, Johnson SJ, Draper ES, Costeloe KL, Marlow N. Neurological and developmental outcome in extremely preterm children born in England in 1995 and 2006: the EPICure studies. *British Medical Journal*. 2012. 345: e7961.
18. Horby P, Lim WS, Emberson JR, Mafham M, Bell JR, Linsell L, Staplin N, Brightling C, Ustianowski A, Elmahi E, Prudon B, Green C, Felton T, Chadwick D, Rege K, Fegan C, Chappell LC, Faust SN, Jaki T, Jeffery K, Montgomery A, Rowan K, Juszxzak E, Baillie JK, Haynes R, Landray MJ on behalf of the RECOVERY Collaborative Group.

For peer review only - http://bmjopen.bmj.com/site/about/guidelines.xhtml

Dexamethasone in Hospitalized Patients with Covid-19. *New England Journal of Medicine*. 2021. 384(8): 693-704.

Figure Legend:

Figure 1: Example PICO from PlaNeT-2 Trial

Figure 2: Steering Group Members

For peer review only

Example PICO from PlaNeT-2 Trial (Platelet transfusion thresholds in premature neonates)[18]

Participants: Preterm Infants < 34 weeks gestation at birth

Intervention: Low transfusion threshold (transfusing patient if platelet count < $25x10^9$/L)

Control: High transfusion threshold (transfusing patient if platelet count <$50x10^9$/L)

Outcome: Primary Outcomes were mortality or major bleeding within 28 days. (Secondary Outcomes were bronchopulmonary dysplasia, sepsis, retinopathy of prematurity and necrotising enterocolitis.)

For peer review only

**Steering Group Member**	**Role and affiliation**
Cheryl Battersby (CB)	Academic Neonatologist, BAPM Data/Informatics lead & member of NIHR prioritisation committee.
James Boardman (JB)	Professor of Neonatal Medicine and immediate past president of the Neonatal Society.
William Carroll (WC)	Consultant Paediatrician and RCPCH officer for Research.
Jon Dorling (JD)	Professor of Paediatrics, Neonatal Consultant and BAPM research lead.
Kate Dinwiddy (KD)	Chief Executive of BAPM.
Katie Evans (KE)	Project Co-ordinator and Honorary Clinical Research Fellow in Neonatal Medicine.
Chris Gale (CG)	Academic Neonatologist and Neonatal Society Meeting Secretary.
Katie Gallagher (KG)	Academic Neonatal Nurse and Neonatal Nurses Association representative.
Pollyanna Hardy (PH)	Clinical Trials Statistician and Director of National Perinatal Epidemiology Unit.
Emma Johnston (EJ)	Parent representative and Parents and Family engagement Lead with the Thames Valley and Wessex ODN.
Helen Mactier (HM)	Consultant Neonatologist, Honorary Clinical Associate Professor and President of BAPM.
Claire Marcroft (CM)	Neonatal Physiotherapist and Allied Health Professionals Representative.
James Webbe (JW)	Trainee representative and Neonatal Medicine GRID Trainee.

For peer review only

BMJ Open

Protocol: A national priority setting partnership using a Delphi consensus process to develop neonatal research questions suitable for practice-changing randomised trials in the United Kingdom

Journal:	*BMJ Open*
Manuscript ID	bmjopen-2022-061330.R1
Article Type:	Protocol
Date Submitted by the Author:	23-Jul-2022
Complete List of Authors:	Evans, Katie; Imperial College London Faculty of Medicine,<br>Battersby, Cheryl; Imperial College London, NHS England<br>Boardman, James; The University of Edinburgh, MRC Centre for Reproductive Health<br>Boyle, Elaine; University of Leicester, Health Sciences<br>Carroll, William; University Hospitals of North Midlands NHS Trust, Academic Department of Child Health; Keele University, Institute of Applied Clinical Sciences<br>Dinwiddy, Kate; British Association of Perinatal Medicine<br>Dorling, Jon; University Hospital Southampton NHS Foundation Trust<br>Gallagher, Katie; University College London EGA Institute for Women's Health, Child and Adolescent Health<br>Hardy, Pollyanna; University of Oxford, Clinical Trials<br>Johnston, Emma; Parents and Families engagement lead at Thames Valley and Wessex Operational Deliveries Network<br>Mactier, Helen; University of Glasgow<br>Marcroft, Claire; Newcastle Upon Tyne Hospitals NHS Foundation Trust<br>Webbe, James; Imperial College London, Section of Neonatal Medicine<br>Gale, Chris; Imperial College London, Neonatal Medicine, School of Public Health
**Primary Subject Heading**:	Paediatrics
Secondary Subject Heading:	Paediatrics
Keywords:	NEONATOLOGY, Protocols & guidelines < HEALTH SERVICES ADMINISTRATION & MANAGEMENT, Neonatal intensive & critical care < INTENSIVE & CRITICAL CARE

SCHOLARONE™
Manuscripts

Protocol: A national priority setting partnership using a Delphi consensus process to develop neonatal research questions suitable for practice-changing randomised trials in the United Kingdom.

Authors:

Katie Evans, BSc (hons), MBBS, MRCPCH. Honorary research fellow, Neonatal Medicine, Imperial College London. E: katie.evans7@nhs.net.

Cheryl Battersby, PhD. Neonatal Medicine, Imperial College London, School of Public Health, Faculty of Medicine. E: c.battersby@imperial.ac.uk.

James P. Boardman, PhD, FRCPCH. Professor of Neonatal Medicine, MRC Centre for Reproductive Health, University of Edinburgh, UK. E: james.boardman@ed.ac.uk.

Elaine Boyle, PhD, MD. Professor of Neonatal Medicine, University of Leicester. Chair of the National Institute for Health Research Neonatal Clinical Studies Group.

William Carroll, MD, FRCPCH. Honorary Reader in Child Health, Keele University, Keele, UK. E: will.carroll@nhs.net.

Kate Dinwiddy, BA (hons). Chief Executive of British Association of Perinatal Medicine. E: kate.dinwiddy@rcpch.ac.uk.

Jon Dorling, MBChB, MD, FRCPCH. Department of Neonatal Medicine. Consultant Neonatology and Adjunct Professor. University Hospital Southampton NHS Foundation Trust, Southampton, UK. E: jon.dorling@uhs.nhs.uk.

Katie Gallagher, PhD, RN (child), Senior Research Fellow, EGA Institute for Women's Health, University College London, UK. E: katie.gallagher@ucl.ac.uk.

Pollyanna Hardy, MSc. Director of the National Perinatal Epidemiology Unit Clinical Trials Unit, University of Oxford, UK. E: Pollyanna.hardy@npeu.ox.ac.uk.

For peer review only - http://bmjopen.bmj.com/site/about/guidelines.xhtml

Emma Johnston, BA (hons), DipM. Parents and Family engagement Lead with the Thames Valley and Wessex Operational Delivery Network, UK. E: emma_jj@outlook.com.

Helen Mactier, MBChB, MD, FRCPCH. Consultant Neonatologist and Honorary Clinical Associate Professor, University of Glasgow. E: helen.mactier@glasgow.ac.uk.

Claire Marcroft, Clinical Specialist Neonatal Physiotherapist and NIHR CDRF Fellow, Newcastle upon Tyne Hospitals NHS Foundation Trust.

James Webbe, PhD, Neonatal Medicine, School of Public Health, Faculty of Medicine, Imperial College London. E: j.webbe@imperial.ac.uk.

Chris Gale, PhD, Reader in Neonatal Medicine, Imperial College London, School of Public Health, Faculty of Medicine, Chelsea and Westminster campus, 4th Floor, Lift Bank D, 369 Fulham Road, SW10 9NH, UK; Telephone: 0203 3153519; E: christopher.gale@imperial.ac.uk (corresponding author)

For peer review only

For peer review only - http://bmjopen.bmj.com/site/about/guidelines.xhtml

Abstract:

Introduction. Methodologically robust clinical trials are required to improve neonatal care and reduce unwanted variations in practice. Previous neonatal research prioritisation processes have identified important research themes rather than specific research questions amenable to clinical trials. Practice-changing trials require well defined research questions, commonly organised using the Population, Intervention, Comparison, Outcome (PICO) structure. By narrowing the scope of research priorities to those which can be answered in clinical trials and by involving a wide range of different stakeholders, we aim to provide a robust and transparent process to identify and prioritise research questions answerable within the NHS to inform future practice-changing clinical trials.

Methods and Analysis. A steering group comprising parents, doctors, nurses, allied health professionals, researchers, and representatives from key organisations (Neonatal Society (NS), British Association of Perinatal Medicine (BAPM), Neonatal Nurses Association (NNA) and Royal College of Paediatrics and Child Health (RCPCH)) was identified to oversee this project. We will invite submissions of research questions formatted using the PICO structure from the following stakeholder groups using an online questionnaire: parents, patients, healthcare professionals and academic researchers. Unanswered, non-duplicate research questions will be entered into a three round eDelphi survey of all stakeholder groups. Research questions will be ranked by mean aggregate scores.

Ethics and Dissemination. The final list of prioritised research questions will be disseminated through traditional academic channels, directly to key stakeholder groups through representative organisations and on social media. The outcome of the project will be shared with key research organisations such as the National Institute for Health Research (NIHR). Research ethics committee approval is not required.

Registration Details. Not registered.

For peer review only

Strengths and Limitations of this study:

- By involving parents and former patients alongside a wide range of healthcare professionals we will ensure that research questions are important to all key stakeholder groups.
- We will use established strategies (three round eDelphi process) to rank research questions based upon mean scores.
- The study will rank research questions based upon subjective input from a large number of key stakeholders, however questions may need further work prior to being addressed in a clinical trial.
- The final list of prioritised questions will be disseminated widely to inform neonatal clinical research.

For peer review only

Introduction:

The importance of involving different stakeholders including parents, patients, healthcare professionals and researchers to identify and prioritise research is well recognised[1]. In neonatal and perinatal medicine these stakeholder groups have prioritised research uncertainties in the fields of preterm birth[2,3], stillbirth[4], diabetes in pregnancy[5] and pregnancy hypertension[6]. Although these processes have been invaluable for prioritising research themes, the broad topics commonly identified have not been readily amenable to testing in high-quality interventional studies. Methodologically robust randomised controlled trials (RCTs) are the gold standard for assessing effectiveness of a healthcare intervention, drug or technology[7] and are critical to improving quality and reducing variation in neonatal care. Interventional studies such as RCTs require clearly structured and focused research questions, which can be organised using the PICO model which guides the questioner to clearly identify the Participant (P), Intervention (I), Comparator (C) and Outcome (O)[8,9] (figure 1)[10].

There is wide variation in neonatal care[11,12] and an incomplete evidence base for many neonatal treatments.[13] Consequently, there is a need to identify and prioritise research questions that can be tested in randomised trials. Involving all relevant stakeholders in such a process will ensure that research addresses questions that are important to healthcare professionals, former neonatal patients and parents, as well as relevant to current neonatal care.

The aim of this project is to identify and prioritise neonatal research questions in PICO format, suitable for high-quality interventional studies, using a robust, transparent, and inclusive methodology. The results of this identification and prioritisation process will inform the development and design of neonatal interventional studies in high income settings, including the National Healthcare System (NHS) in the United Kingdom.

Methods and Analysis

Steering Group

A steering group was formed in September 2021 to agree the scope and facilitate the process of identifying and prioritising research questions. The following key stakeholders are represented on the steering group: the Neonatal Society (NS), British Association of Perinatal Medicine (BAPM), Neonatal Nurses Association (NA), Royal College of Paediatrics and Child Health, neonatal Allied Health Professionals (AHPs), neonatal clinical trial methodologists,

For peer review only - http://bmjopen.bmj.com/site/about/guidelines.xhtml

neonatal trainees and parents with experience of neonatal care. The steering group is co-chaired by representatives of the NS and BAPM and has an allocated project co-ordinator responsible for agenda, minuting, role allocation and participant support Details of the steering group members and their affiliations can be found in figure 2.

The roles of the steering group are as follows:

- Agreeing the scope of the process.
- Disseminating details of the process.
- Engaging with clinical stakeholders to take part in identifying and prioritising research questions.
- Review of submitted research questions to identify duplicate questions, questions that have already been answered and questions outside the scope.
- Disseminating the final ranked list of research questions.

Identification of stakeholders

The utility and validity of this project will depend on ensuring that representative questions are generated and prioritised by a wide group of neonatal stakeholders including:

- Clinicians involved in neonatal care: Neonatologists, paediatricians, neonatal nurses, advanced neonatal nurse practitioners (ANNPs) working in paediatrics or neonatal medicine. Recruitment will be through advertisements on the RCPCH website and through other relevant professional organisations including BAPM, the NNA and the NS. Trainee doctors and nurses will be additionally contacted through local training schools and use of communication methods such as regional teaching and social media channels.
- Allied Health Professionals: Occupational therapists, physiotherapists, dieticians, speech and language therapists and clinical psychologists working in neonatal care: Advertisements will be placed through professional websites and organisations and co-ordinated by the AHP steering group representative.
- Academics and researchers working within neonatology: Recruitment will be targeted through academic organisations, existing research networks, national meetings and through Clinical Trials Units with a neonatal interest.

For peer review only - http://bmjopen.bmj.com/site/about/guidelines.xhtml

- Parents and former neonatal patients: These groups will be contacted through the National Care Coordinator Group, Maternity Voices partnerships, charity websites and through social media platforms.

To ensure maximal engagement across all stakeholder groups, various communications strategies will be employing including email contact, professional websites and social media routes.

Patient and Public Involvement

Parents and patients have been involved in this work from its inception. A parent representative sits on the steering group to plan the project and optimise parent and patient involvement. Communication to parents and former neonatal patients will be through parent and patient networks charities, regional support networks and social media groups. We recognise the importance of hearing from minority ethnic communities and those from low socio-economic backgrounds, particularly in view of their greater risks of pregnancy complications that may lead to experience of neonatal care[14]. These stakeholders were contacted directly through relevant parent-led and charitable organisations with requests to share details of the project on their social media platforms and email lists. The communications strategy prioritises working with a wide range of people from all backgrounds and plans to ensure accessible language translations of research questions during the final parental and former patient prioritsation process.

Parents and former neonatal patients will be involved in identifying neonatal research questions through the online identification process. We acknowledge that there will need to be extensive support for non-academic participants to ensure they are not dissuaded by the PICO structure. To further facilitate the identification of research questions from parents and former neonatal patients we will develop targeted online support resources and run several online workshops focused on developing questions using a PICO format. We will develop a training video targeted specifically towards parents and former patients and develop our resource page on the BAPM website with further written information about formatting a PICO question. In addition our question building software will be designed specifically to walk participants through the PICO process, with pre-formatted options for populations and outcomes to make it more accessible.

For peer review only - http://bmjopen.bmj.com/site/about/guidelines.xhtml

Parents and former neonatal patients will then be invited to participate in the prioritisation of research questions through the eDelphi process. Due to the nature of the study, some questions may require a high level of medical understanding, and so there will be the option for "unable to rank" if a participant feels that they cannot make a meaningful judgement on assessing its importance. Throughout the eDelphi we will seek feedback from parents and former patients regarding its accessibility. Balancing meaningful parental involvement with the specificity required for questions to be answered in interventional trials may be challenging. Therefore with the results of the final eDelphi survey we will carry out a specific targeted prioritisation process with parents, families and former patients, with support from Bliss, to ensure that this important group of voices are heard.

Scope

The scope of the prioritisation process has been agreed by the steering group as follows:

- Limited to research questions relevant to high income neonatal care settings.
- Limited to research questions related to care provided by neonatal teams:
  - On neonatal units, transitional care units, or as part of neonatal transport.
  - On postnatal wards (excluding care exclusively provided by midwifery teams without neonatal input).
  - In the community after receiving neonatal care as an inpatient (care may be provided by community neonatal teams or associated community allied health professionals with neonatal interest).

Overview

This study will be divided into four stages which will commence in January 2022 and complete by September 2022 (figure 3):

1. Identification of neonatal research questions suitable for analysis in randomised controlled trials. (1 month)
2. Review of submitted neonatal research questions to remove duplicate questions and previously answered questions. (1 month)
3. Prioritisation of neonatal research questions by all relevant stakeholders using a three-round eDelphi process. (3 months)
4. Dissemination of ranked list of PICO questions.

For peer review only - http://bmjopen.bmj.com/site/about/guidelines.xhtml

Stage 1: Identification of testable neonatal research questions

We will identify neonatal research questions suitable for evaluation in practice changing randomised controlled trials through an open process. Individual stakeholders (neonatal clinicians, neonatal nurses, ANNPs, neonatal AHPs, neonatal researchers and former neonatal patients and patients) will be contacted via professional organisations, social media platforms, networks and organisational mailing lists. The process for identifying research questions will also be openly publicised on organisational websites seeking submission of research questions from stakeholders. Publicity will precede and continue throughout a four-week submission period[15] to optimise engagement and inclusion of all key groups.

Stakeholders will be invited to submit as many questions as they would like using an online system that facilitates submission using the PICO structure:

- Populations will be able to be selected from a pre-defined selection of gestations and clinical cohorts, or specified by the submitter
- Intervention and Comparisons will take the form of free text to allow full descriptions
- Outcomes will be able to be selected from Core Outcomes in Neonatology (COIN)[16], or specified by the submitter to ensure both core outcomes and individual outcomes are valued equally.

Support in structuring research questions using PICO will be facilitated by the steering group members and organisations using techniques such as short videos and webinars. Contact details and basic demographic data will be requested from individuals who submit questions to monitor the representativeness of stakeholder involvement, and to invite participation in the subsequent prioritisation process.

Submission of relevant PICOs from previous priority setting work[17] will be welcomed.

Stage 2: Review and Refinement of the long-list of neonatal research questions

Submitted research questions will be reviewed by the steering group to remove duplicate, already answered and out of scope questions, and to refine those not consistent with the PICO structure. Each question will be independently reviewed by two separate members of the steering group to ensure a transparent and reproducible process. It is recognised that there can be widely variable views on whether a clinical question has been adequately 'answered' by

For peer review only

existing research and in view of this any questions excluded on this rationale will have to receive consensus view from all members of the steering group. Prior to exclusion, questions will be independently reviewed by two members of the steering group. A final long-list of research questions will be taken forward for prioritisation.

<u>Stage 3: Prioritisation of Neonatal Research Questions</u>

All research questions included in the long-list will be entered into in a three-round eDelphi prioritisation process. Involvement of stakeholders in the prioritisation process will be facilitated as follows:

- All stakeholders who submitted questions will be contacted by email and asked to take part.
- Invitations to take part will be circulated by professional organisations and open links will be made available on professional organisational websites and social media accounts.
- Parents and previous neonatal patients will be contacted through professional and charitable organisations and networks and through social media.

All research questions will be ranked using a 9-point Likert scale. After the first round there will be the opportunity to submit new PICO questions. These additional questions will pass through the same two stage review process, co-ordinated by the steering group as the original questions. Prior to opening the second round of the Delphi survey, a steering group meeting will define the threshold for questions to take part in this round.During the second round, participants will be provided with information on how individual research questions were prioritised by each stakeholder group during the first round. Participants will then be able to amend or confirm their original ratings, taking into consideration other stakeholder group opinions. Prior to the third round the steering group will convene a meeting and review the preliminary results to decide the threshold of questions to enter the third round. A shorter list of questions in the third round will help to improve retention of participants by facilitating a shorter time commitment.

After completion of the third round of ranking all research questions entered into the Delphiwill be collated into a ranked list of research priorities for the UK neonatal community. Ranking will be ordered based upon mean score. Results will also be presented by mean score within

For peer review only - http://bmjopen.bmj.com/site/about/guidelines.xhtml

each stakeholder group. All questions not excluded in the initial review process will be included in the final list.

Ethics and Dissemination:

Research Ethics Committee approval is not required for this work.

The ranked list of defined neonatal research questions will be disseminated as follows:

- We will share results directly with the NIHR prioritisation panel and other funders of neonatal trials.
- We will circulate the list to stakeholder organisations and their members.
- Individuals who participated in question setting or the prioritisation process will be emailed directly with the finalised rankings and named as group authors in any published work.
- Results will be disseminated amongst the scientific community through a publication and presented at relevant neonatal meetings.

Discussion:

More than 1 in 10 babies in many high-income settings will receive neonatal care. Neonatal conditions are important, contributing to almost half of all child deaths in the UK and to many long-term health conditions. Despite this importance, much neonatal care is not based upon high-quality research evidence. Historically, the perinatal research environment has fostered collaborative working, demonstrated by studies such as EPICure[18]. The SARS-CoV-2 pandemic has further highlighted the benefits of joined up working across multiple geographical regions, scientific institutions and research groups in achieving practice changing outcomes within much shorter timeframes[19]. This has resulted in increased focus on prioritisation projects within healthcare using initiatives such as the James Lind Alliance to support unified decision making across specific specialities. Neonatology within the UK is ideally placed for such prioritisation work and future collaborative research due to centrally funded healthcare, the neonatal operational delivery network structure facilitating close relationships between units and the accessibility of large national databases. Ultimately this process seeks to involve a wide range of key neonatal stakeholders, to identify and prioritise

For peer review only - http://bmjopen.bmj.com/site/about/guidelines.xhtml

research questions addressing the many clinical uncertainties, suitable for evaluating in well-structured clinical trials.

Author Contributions:

CG and CB conceived this project. KE, JPB, HM, CG and CB planned and coordinated the initial steering group and protocol. KD provided administrative support. The first draft of the manuscript was written by KE and revised by CB/ JPB/ WC/ JD/ KG/ PH/ EJ/ HM/ JW and CG. CG edited and reviewed the manuscript. It was approved by all members of the steering group.

Correspondence to Chris Gale, PhD, Reader in Neonatal Medicine, Imperial College London, School of Public Health, Faculty of Medicine, Chelsea and Westminster campus, 4th Floor, Lift Bank D, 369 Fulham Road, SW10 9NH, UK; Telephone: 0203 3153519; E: christopher.gale@imperial.ac.uk.

Funding Statement:

Administrative support for the project is provided by BAPM; questionnaire and online Delphi software are funded by the Medical Research Council (MRC) through a Transition Support Award held by CG (MR/V036866/1).

Competing Interests Statement:

CG is vice chair of the NIHR Research for Patient Benefit London Regional Advisory Panel and a member of the Glasgow Children's Hospital Charity External Panel; he holds a Medical Research Council Transition Support Award.
JPB is a member of the Wellcome Trust's Career Development Award Panel and the Great Ormond Street Hospital Charity Research Assessment Panel.
CB is the NIHR deputy chair of HTA prioritisation committee for hospitals.
JD is a member of the NIHR HTA CET Funding Committee.
CM is funded by HEE-NIHR Integrated Clinical Academic Programme and holds a NIHR ICA CSRF Fellowship.

For peer review only - http://bmjopen.bmj.com/site/about/guidelines.xhtml

Acknowledgements:

We wish to thank the members of the Neonatal Priority Setting Partnership Steering Group for their support and RCPCH, BAPM, NNA and the NS for their support.

Word Count:

2616/ 4000 (including abstract/ excluding references)

References

1. Partridge N, Scadding J. The James Lind Alliance: patients and clinicians should jointly identify their priorities for clinical trials. *Lancet.* 2004; 364(9449): 1923-1924.
2. Duley L, Uhm S, Oliver S, Par PBPS. Top 15 UK research priorities for preterm birth. *Lancet.* 2014. 383(9934): 2041-2042.
3. Oliver S, Uhm S, Duley L, Crowe S, David AL, James CP, Chivers Z, Gyte G, Gale C, Turner M, Chambers B, Dowling I, McNeill J, Alderdice F, Shennan A & Deshpande S. Top research priorities for preterm birth: results of a prioritisation partnership between people affected by preterm birth and healthcare professionals. *BMC Pregnancy Childbirth.* 2019. 19(1): 528.
4. Heazell AEP, Whitworth MK, Whitcombe J, Glover SW, Bevan C, Brewin J, Calderwood C, Canter A, Jessop F, Johnson G, Martin I, Metcalf L. Research Priorities for Stillbirth: Process Overview and Results from the UK Stillbirth Priority Setting Partnership. *Ultrasound Obstetrics Gynaecology.* 2015. 46(6):641-647.
5. Ayman G, Strachan JA, McLennan N, Malouf R, Lowe-Zinola J, Magdi F, Roberts N, Alderdice F, Berneantu I, Breslin N, Byrne C, Carnell S, Churchill D, Grisoni J, Hirst JE, Morris A, Murphy HR, O'Brien J, Schmutz C, Shah K, Singal AS, Strachan MWJ, Cowan K, Knight M. The top 10 research priorities in diabetes and pregnancy according to women, support networks and healthcare professionals. *Diabetic Medicine.* 2021. 38(8):e14588.
6. Ho A, Webster L, Bowen L, Creighton F, Findlay S, Gale C, Green M, Gronlund T, Magee L, McManus RJ, Mistry HD, Singleton G, Thornton J, Whybrow R, Chappell L. Research priorities for pregnancy hypertension: a UK priority setting partnership with the James Lind Alliance. *BMJ Open.* 2020. 10:e036347.

For peer review only - http://bmjopen.bmj.com/site/about/guidelines.xhtml

7. Akobeng AK. Understanding randomised controlled trials. *Archives of Disease in Childhood.* 2005. 90:840-844.
8. Miller SA, Forrest JL. Enhancing your practice through evidence-based decision making: PICO, learning how to ask good questions. *Journal of Evidence Based Dental Practice.* 2001. 1: 136-141.
9. Richardson WS, Wilson MC, Nishikawa J, Hayward RS. The well-built clinical question: a key to evidence-based decisions. *ACP Journal Club.* 1995. 123(3): 12-13.
10. Curley A, Stanworth SJ, Willoughby K, Fustolo-Gunnink SF, Venkatesh V, Hudson C, Deary A, Hodge R, Hopkins V, Santamaria BL, Mora A, Llewelyn C, A-Amore A, Khan R, Onland W, Lopriore E, Fijnvandraat K, New H, Clarke P, Watts T on behalf of the PlaNeT2 MATISSE Collaborators. Randomized Trial of Platelet-Transfusion Thresholds in Neonates. *New England Journal of Medicine.* 2019. 380: 242-251.
11. National Neonatal Audit Programme (NNAP) 2020 annual report on 2019 data. RCPCH. London, 2020.
12. Pierrat V, Burguet A, Marchand-Martin L, Cambonie G, Coquelin A, Roze JC, Durox M, Guillois B, Morgan AS, Kaminski M on behalf of the Neurodevelopmental care study group of EPIPAGE-2. Variations in patterns of care across neonatal units and their associations with outcomes in very preterm infants: the French EPIPAGE-2 cohort study. *BMJ Open.* 2020. 10:e035075.
13. Willhelm C, Girisch W, Gottschling S, Graber S, Wahl H, Meyer S. Systematic Cochrane reviews in neonatology: a critical appraisal. *Paediatric Neonatology.* 2013. 54(4): 261-266.
14. Draper ES,Gallimore ID, Smith LK, Fenton AC, Kurinczuk JJ, Smith PW, Boby T, Manktelow BD on behalf of the MBRRACE-UK Collaboration. MBRACE-UK Perinatal Mortality Surveillance Report, UK Perinatal Deaths for Births from January to December 2018. *Department of Health Sciences, University of Leicester*. 2020.
15. Kost RG, Rosa JCd. Impact of survey length and compensation on validity, reliability and sample characteristics for Ultrashort-, Short-, and Long-Research Participant Perception Surveys. *Clinical Translational Science.* 2018. 2(1): 242-251.
16. Webbe J, Duffy JMN, Afonso E, Al-Muzaffar I, Brunton G, Greenough A, Hall NJ, Knight MK, Latour JM, Lee-Davey C, Marlow N, Noakes L, Nycyk J, Richard-Londt A, Wills-Eve B, Modi N, Gale C. Core outcomes in neonatology: development of a core outcome set for neonatal research. *Archives of Disease in Childhood - Fetal and Neonatal Edition.* 2020. **105:** 425-431.

For peer review only - http://bmjopen.bmj.com/site/about/guidelines.xhtml

17. Johnson MJ, Lapillonne A, Bronsky J, Domellof M, Embleton N, Iacobelli S, Jochum F, Joosten K, Kolacek S, Mihatsch WA, Moltu SJ, Puntis JWL, Riskin A, Shamir R, Tabbers MM, Goudoever JBV, Pipaon MSd on behalf of the ESPGHAN/ ESPEN/ ESPR/ CSPEN Working Group on Pediatric Parenteral Nutrition. Research priorities in pediatric parenteral nutrition: a consensus and perspective from ESPGHAN/ ESPEN/ ESPR/ CSPEN. *Pediatric Research.* 2021. Epub ahead of print: doi:10.1038/s41390-021-01670-9.
18. Moore T, Hennessy EM, Myles J, Johnson SJ, Draper ES, Costeloe KL, Marlow N. Neurological and developmental outcome in extremely preterm children born in England in 1995 and 2006: the EPICure studies. *British Medical Journal*. 2012. 345: e7961.
19. Horby P, Lim WS, Emberson JR, Mafham M, Bell JR, Linsell L, Staplin N, Brightling C, Ustianowski A, Elmahi E, Prudon B, Green C, Felton T, Chadwick D, Rege K, Fegan C, Chappell LC, Faust SN, Jaki T, Jeffery K, Montgomery A, Rowan K, Juszxzak E, Baillie JK, Haynes R, Landray MJ on behalf of the RECOVERY Collaborative Group. Dexamethasone in Hospitalized Patients with Covid-19. *New England Journal of Medicine.* 2021. 384(8): 693-704.

Figure Legend:

Figure 1: Example PICO from PlaNeT-2 Trial

Figure 2: Steering Group Members

Figure 3: Methods

For peer review only

For peer review only - http://bmjopen.bmj.com/site/about/guidelines.xhtml

Example PICO from PlaNeT-2 Trial (Platelet transfusion thresholds in premature neonates)[18]

Participants: Preterm Infants < 34 weeks gestation at birth

Intervention: Low transfusion threshold (transfusing patient if platelet count < $25x10^9/L$)

Control: High transfusion threshold (transfusing patient if platelet count <$50x10^9/L$)

Outcome: Primary Outcomes were mortality or major bleeding within 28 days. (Secondary Outcomes were bronchopulmonary dysplasia, sepsis, retinopathy of prematurity and necrotising enterocolitis.)

For peer review only

Steering Group Member	Role and affiliation
Cheryl Battersby (CB)	Academic Neonatologist, BAPM Data/Informatics lead & member of NIHR prioritisation committee.
James Boardman (JB)	Professor of Neonatal Medicine and immediate past president of the Neonatal Society.
Elaine Boyle (EB)	Professor of Neonatal Medicine and Chair of the National Institute for Health Research Neonatal Clinical Studies Group.
William Carroll (WC)	Consultant Paediatrician and RCPCH officer for Research.
Jon Dorling (JD)	Professor of Paediatrics, Neonatal Consultant and BAPM research lead.
Kate Dinwiddy (KD)	Chief Executive of BAPM.
Katie Evans (KE)	Project Co-ordinator and Honorary Clinical Research Fellow in Neonatal Medicine.
Chris Gale (CG)	Academic Neonatologist and Neonatal Society Meeting Secretary.
Katie Gallagher (KG)	Academic Neonatal Nurse and Neonatal Nurses Association representative.
Pollyanna Hardy (PH)	Clinical Trials Statistician and Director of National Perinatal Epidemiology Unit.
Emma Johnston (EJ)	Parent representative and Parents and Family engagement Lead with the Thames Valley and Wessex ODN.
Helen Mactier (HM)	Consultant Neonatologist, Honorary Clinical Associate Professor and President of BAPM.
Claire Marcroft (CM)	Neonatal Physiotherapist and Allied Health Professionals Representative.
James Webbe (JW)	Trainee representative and Neonatal Medicine GRID Trainee.

For peer review only

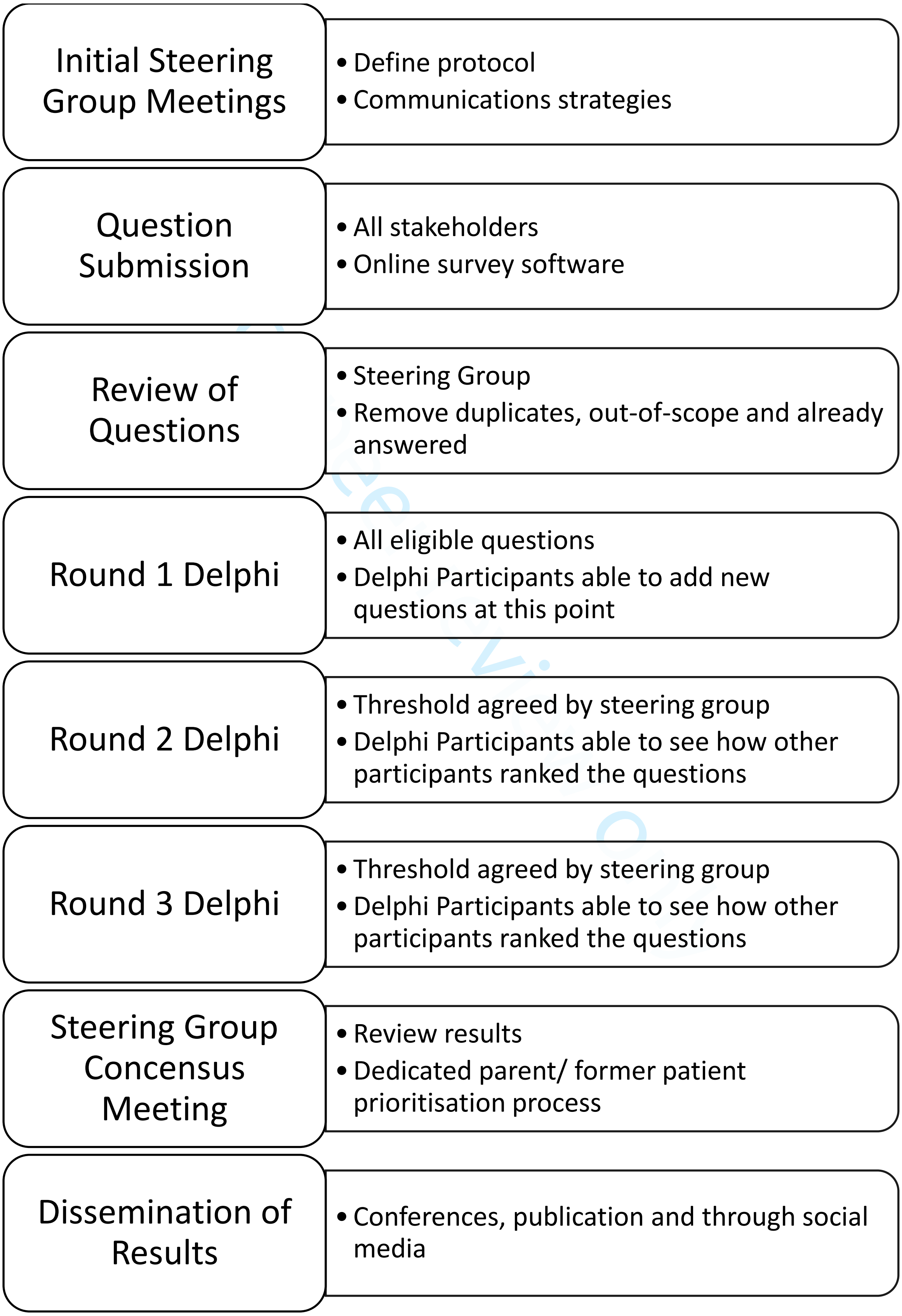
Initial Steering Group Meetings
• Define protocol
• Communications strategies
Question Submission
• All stakeholders
• Online survey software
Review of Questions
• Steering Group
• Remove duplicates, out-of-scope and already answered
Round 1 Delphi
• All eligible questions
• Delphi Participants able to add new questions at this point
Round 2 Delphi
• Threshold agreed by steering group
• Delphi Participants able to see how other participants ranked the questions
Round 3 Delphi
• Threshold agreed by steering group
• Delphi Participants able to see how other participants ranked the questions
Steering Group Concensus Meeting
• Review results
• Dedicated parent/ former patient prioritisation process
Dissemination of Results
• Conferences, publication and through social media
